# Expression of ligands for activating natural killer cell receptors on cell lines commonly used to assess natural killer cell function

**DOI:** 10.1186/s12865-018-0272-x

**Published:** 2019-01-29

**Authors:** Alexandra Tremblay-McLean, Sita Coenraads, Zahra Kiani, Franck P. Dupuy, Nicole F. Bernard

**Affiliations:** 1 Research Institute of the McGill University Health Center, Glen Site, 1001 Décarie Boulevard, Block E, Rm EM3.3238, Montréal, Québec H4A 3J1 Canada; 2 Division of Experimental Medicine, McGill University, Montréal, Québec Canada; 3 Chronic Viral Illness Service, McGill University Health Centre, Montréal, Québec Canada; 4 Division of Clinical Immunology, McGill University Health Centre, Montréal, Québec Canada

**Keywords:** NK cells, Activating NK receptor ligands, HLA null cells, K562, 721.221, CEM.NKr.CCR5

## Abstract

**Background:**

Natural killer cell responses to virally-infected or transformed cells depend on the integration of signals received through inhibitory and activating natural killer cell receptors. Human Leukocyte Antigen null cells are used in vitro to stimulate natural killer cell activation through missing-self mechanisms. On the other hand, CEM.NKr.CCR5 cells are used to stimulate natural killer cells in an antibody dependent manner since they are resistant to direct killing by natural killer cells. Both K562 and 721.221 cell lines lack surface major histocompatibility compatibility complex class Ia ligands for inhibitory natural killer cell receptors. Previous work comparing natural killer cell stimulation by K562 and 721.221 found that they stimulated different frequencies of natural killer cell functional subsets. We hypothesized that natural killer cell function following K562, 721.221 or CEM.NKr.CCR5 stimulation reflected differences in the expression of ligands for activating natural killer cell receptors.

**Results:**

K562 expressed a higher intensity of ligands for Natural Killer G2D and the Natural Cytotoxicity Receptors, which are implicated in triggering natural killer cell cytotoxicity. 721.221 cells expressed a greater number of ligands for activating natural killer cell receptors. 721.221 expressed cluster of differentiation 48, 80 and 86 with a higher mean fluorescence intensity than did K562. The only ligands for activating receptor that were detected on CEM.NKr.CCR5 cells at a high intensity were cluster of differentiation 48, and intercellular adhesion molecule-2.

**Conclusions:**

The ligands expressed by K562 engage natural killer cell receptors that induce cytolysis. This is consistent with the elevated contribution that the cluster of differentiation 107a function makes to total K562 induced natural killer cell functionality compared to 721.221 cells. The ligands expressed on 721.221 cells can engage a larger number of activating natural killer cell receptors, which may explain their ability to activate a larger frequency of these cells to become functional and secrete cytokines. The few ligands for activating natural killer cell receptors expressed by CEM.NKr.CCR5 may reduce their ability to activate natural killer cells in an antibody independent manner explaining their relative resistance to direct natural killer cell cytotoxicity.

**Electronic supplementary material:**

The online version of this article (10.1186/s12865-018-0272-x) contains supplementary material, which is available to authorized users.

## Background

Natural Killer (NK) cells are a subset of lymphocytes that direct innate immune responses to kill stressed, virally-infected, and transformed cells [1]. NK cells belong to the group 1 innate lymphoid cells (ILCs) as do ILC1s [2–4]. NK cells can interact with target cells and lyse them directly via the release of cytotoxic granules containing perforin and granzyme [5, 6]. Activated NK cells can also secrete a broad range of cytokines and chemokines that can activate adaptive immune cells to lyse target cells, bridging the innate and adaptive immune systems [7–9]. Regardless of the method used by NK cells to lyse target cells, they must first be activated to elicit a response. The activation state of an NK cell results from the integration of different signals transmitted through its activating and inhibitory natural killer cell receptors (aNKR and iNKR) [9, 10]. Activation can result from the loss of inhibitory signaling, when there are no ligands for iNKR to engage, in conjunction with sustained aNKR signaling, or from the engagement of aNKR by their ligands that overwhelms inhibitory signaling through iNKR [8, 10, 11]. However, in vivo, NK cells interacting with target cells will receive a variety of signals through both receptor types and whether this results in activation or not depends on the number and strength of each type of signal transmitted.

A common method to activate NK cells is to co-culture them with human leukocyte antigen (HLA)-null cells. The most frequently used HLA-null cells include the myelogenous leukemia K562 and B-lymphoblastoid 721.221 (.221) cells lines, which do not express the major histocompatibility complex class I (MHC-I) HLA-A, -B, or -C antigens on their surface [12–14]. As these cells are incapable of engaging the inhibitory killer immunoglobulin-like receptors (KIR) on NK cells, which recognize subsets of HLA-A, -B, and -C, inhibitory signaling through these receptors is abrogated. As signals from these inhibitory receptors oppose those from aNKR, their removal allows the engagement of aNKR by their ligands to activate NK cells [15].

Previous work from our lab demonstrated that the K562 and .221 HLA-null cell lines stimulated NK cells differentially to secrete the cytokine interferon-γ (IFN-γ), the chemokine CCL4, and to express the degranulation marker CD107a [16]. Specifically, we observed that .221 activated a greater fraction of NK cells than did K562 and that stimulation with .221 preferentially induced IFN-γ and CCL4 secretion, while K562 more potently stimulated degranulation [16]. In the absence of the expression of the major ligands for iNKR on the surface of K562 and .221, it is likely that ligands to aNKR regulate NK cell activation and differences in their expression profiles might explain their different capacities to activate NK cells. Indeed, the expression of some ligands to aNKR was shown to differ on both cell lines, although these differences remain incompletely characterized [17–22].

CEM.NKr.CCR5 cells are commonly used to stimulate NK cells by antibody dependent cellular cytotoxicity (ADCC) [23, 24]. They were derived from CEM.NKr cells that were selected from the parental CEM T cell line for their resistance to direct NK cell lysis [25]. CEM cells express HLA-A, B and C antigens and the aNKR CD16, whose engagement is important for ADCC activity [25, 26]. The aNKR profile of this cell line is poorly defined.

There are two major classes of aNKR. The first is the C-type lectin NKG2D receptor that binds to a family of MHC-I like molecules expressed on healthy cells only after periods of cellular stress [17, 27]. These include the human cytomegalovirus UL-16 binding proteins (ULBP) and MHC-I related chain (MIC) proteins [19, 28, 29]. The second class of receptors includes the natural cytotoxicity receptors (NCR) NKp30, NKp44, and NKp46, which can bind to membrane-associated heparan sulfate glycosaminoglycans, viral hemagglutinin and β-1,3-glucan [30–40]. Despite these findings, the cellular ligands to NCRs remain incompletely defined.

In addition to the two major aNKR families, signaling through several other receptors can contribute to NK cell activation. These include cluster of differentiation (CD)244/2B4 and the NK-T-B cell antigen (NTB-A), which are CD2 family receptors that engage CD48 to trigger NK cell cytotoxicity [41]. Another receptor, expressed on virtually all NK cells, is the leukocyte adhesion molecule DNAX accessory molecule-1 (DNAM-1) that binds to CD112 (Nectin-2) and CD155 (poliovirus receptor) ligands [18, 42, 43]. Signaling through DNAM-1 can stimulate NK cells when it is co-expressed with the lymphocyte function-associated antigen 1 (LFA-1). LFA-1 can bind the integrins intercellular adhesion molecule (ICAM)-1 and ICAM-2 on target cells, bridging NK and target cells and forming the immunological synapse [44–48]. Additionally, target cells expressing the T cell co-stimulatory B7 molecules CD80 and CD86 can stimulate NK cells [49–51]. The nature of this signaling is still poorly understood, but it has been suggested that NK cell activation by CD80 and CD86 depends on a CD28 variant expressed on NK cells [50, 52].

In contrast to HLA-A, -B, and -C, which signal through iNKRs, the non-classical HLA-E and -F can contribute to NK cell activation through the engagement of aNKRs. Much like its classical MHC-I counterparts, HLA-E interacts with the inhibitory C-type lectin-like receptor NKG2A, which heterodimerizes with CD94, and with the aNKRs NKG2E and -C [53–55]. HLA-F is a more recent addition to the family of ligands to aNKR and has been shown to stimulate degranulation and cytokine production through its binding to KIR3DS1 on NK cells [56–58].

To determine whether the differences observed in the frequency and function of NK cell subsets stimulated by K562, .221 and CEM.NKr.CCR5 cells are related to differences in their expression profiles of ligands to aNKRs, we analyzed the expression of a comprehensive panel of aNKR ligands on these cell lines by multi-parametric flow cytometry. As ligands to inhibitory KIR are not expressed on HLA-null cell lines, it is likely that expression of ligands to aNKRs plays a role in modulating differential NK cell responses to K562 and .221. Although others have proposed that the resistance of CEM.NKr.CCR5 cells to direct NK cell cytolysis is related to changes in cell surface marker expression that occurred when NK cell resistance to cytolysis was selected for, the ligands for aNKR have not been profiled on this cell line [25]. We report here a characterization of the aNKR ligand phenotype of three NK cell stimuli to contribute to a better understand the mechanisms governing their behavior as inducers of NK cell function and targets for NK cells cytolysis.

## Methods

### K562, .221 and CEM.NKr.CCR5 cell lines

K562 cells (American Type Culture Collection, Manassas, VA) .221 cells (a kind gift from Dr. Galit Alter, Harvard University) and CEM.NKr.CCR5 cells (National Institutes of Health [NIH] AIDS Reagent Program, Division of AIDS, NIAID, NIH, from Dr. Alexandra Trkola) were cryopreserved in 10% dimethyl sulfoxide (Sigma-Aldrich, St. Louis, MO) with 90% fetal bovine serum (FBS, Wisent Bio Porducts, St-Jean-Baptist, QC, Canada). K562 and .221 cells were verified to be HLA-null by staining with the pan HLA monoclonal antibody (mAb) W6/32 and a β2-microglobulin specific mAb [58]. CEM.NKr.CCR5 cells were typed for HLA allotypes using sequence based HLA typing methods and found to express HLA allotypes that were consistent with those previously reported for their parental CEM and CEM.NKr cell lines [25, 59]. Cell lines were thawed and cultured in RPMI 1640 medium supplemented with 10% FBS; 2 mM L-glutamine; 50 IU/mL penicillin; 50 mg/mL streptomycin (R10, all from Wisent) for at least one passage before staining. Cells were passaged three times a week and maintained in culture for a maximum of 1 month. Only healthy cells with a high viability were used in staining experiments.

### Antibody staining and acquisition

Cell lines were stained in triplicate on 1 or 2 occasions or in replicates of six on 1 occasion with UV Live/Dead ® Fixable Blue cell stain kit, as per manufacturer’s directions (Thermofisher, Waltham, MA) and were stained with mAbs clones or chimeric proteins specific for cell surface aNKR ligands organized into 4 panels for the purpose of analysis. Table 1 shows for each mAb and chimeric protein used, its aNKR ligand specificity, the aNKR each ligand recognized, the designation of the antibody clone used for staining, its commercial source and to which fluorochrome antibodies or secondary antibodies recognizing chimeric proteins or primary antibodies were conjugated. Panel 1 included antibodies specific for ULBP-1-PerCP (170818), ULBP-2/5/6-APC (165903), ULBP-3-PE (166510), MIC-A-APC (159227), MIC-B-AlexaFluor 700 (236511; all from R&D Systems, Minneapolis, MN), Panel 2 was made up of antibodies to CD48-PE (BJ40), CD80-BV421 (2D10), CD86-PE-Dazzle594 (IT2.2; all from BioLegend, San Diego, CA), CD112-AlexaFluor 700 (610603; R&D), CD155-PE-Cy7 (SKII.4). Panel 3 comprised antibodies to ICAM-1-Pacific Blue (HCD54), and ICAM-2-PE (CBR-1C2/2; both from BioLegend). Cells were also stained with recombinant human IgG1 Fc chimeric proteins NKp30-Fc, NKp44-Fc, and NKp46-Fc (all from R&D) as a part of Panel 3. A final HLA Panel was composed of the fluorochrome conjugated antibody to HLA-E-PE-Cy7 (3D12; BioLegend), as well as an unconjugated recombinant human IgG1 Fc chimera KIR3DS1-Fc (R&D) and mAb 3D11, an unconjugated primary antibody against HLA-F (a kind gift from Dr. Daniel Geraghty, Fred Hutchison Research Institute, Seattle, WA). Briefly, cells were resuspended in a 96-well V-bottomed plate (Sarstedt, Nümbrecht, Germany) at a concentration of 1 × 10^6^ cells per 100 μL of Dulbecco’s phosphate buffered saline (Wisent) and stained with the Live/Dead reagent. TruStain FcX reagent (BioLegend) was used to minimize non-specific Fc receptor interactions and cells were stained with conjugated mAbs from Panel 1, 2, 3, and HLA for 30 min in the dark at room temperature (RT). For staining with any of the recombinant human IgG1 Fc chimeric proteins or unconjugated mAbs 3D11 and 3D12, cells were prepared as previously described, with the exception that they were stained with chimeric proteins or primary mAb on ice for 40 min. After washing, binding of Fc chimeric proteins was detected using a polyclonal anti-human IgG (Fcγ-specific)-PE conjugated secondary antibody (BioSciences), while 3D11 and 3D12 binding was detected using a polyclonal F (ab’)_2_ anti-mouse IgG-APC conjugated secondary antibody (eBioscience) for 20 min on ice. Following staining, all cells were washed and fixed with 2% paraformaldehyde (Santa Cruz, Dallas TX). Between 400,000 and 600,000 events were acquired on an LSRFortessa × 20 within 24 h (BD). Unstained, single stained controls (CompBead; BD), fluorescence minus one (FMO), and secondary antibody alone controls were used for multi-color compensation and gating purposes. An additional isotype control for the secondary antibodies used to detect KIR3DS1-Fc chimeric protein and unconjugated 3D11 mAb binding to HLA-F were also used. As staining with the antibody specific for ULBP-1 generated signals with a low mean fluorescence intensity (MFI), an isotope control for this mAb was also used as a control in addition to the above-mentioned controls. Flow cytometric analysis was performed using FlowJo software version 10 (TreeStar, Ashland OR).

**Table 1:** Antibody/Chimeric Protein Panel Composition

NK cell Receptor ligand specificity	aNKR/iNKR	Clone	Source	Fluorochrome
Panel 1				
ULBP-1	NKG2D	170818	R & D Systems	PerCP
ULBP-2/5/6	NKG2D	165903	R & D Systems	APC
ULBP-3	NKG2D	166510	R & D Systems	PE
MIC-A	NKG2D	159227	R & D Systems	APC
MIC-B	NKG2D	236511	R & D Systems	AlexaFluor 700
Panel 2				
CD48	2B4	BJ40	BioLegend	PE
CD80	Unknown for NK cells	2D10	BioLegend	BV421
CD86	Unknown for NK cells	IT2.2	BioLegend	PE-Dazzle 594
CD112	DNAM-1	610603	R & D Systems	AlexaFluor 700
CD155	DNAM-1	SKII.4	BioLegend	PE-Cy7
Panel 3				
ICAM-1	LFA-1	HCD54	BioLegend	Pacific Blue
ICAM-2	LFA-1	CBR-1C2/2	BioLegend	PE
Unknown	NKp30	NKp30-Fc	R & D Systems	Unconjugated^a^
Unknown	NKp44	NKp44-Fc	R & D Systems	Unconjugated^a^
Unknown	NKp46	NKp46-Fc	R & D Systems	Unconjugated^a^
HLA Panel				
HLA-F	KIR3DS1	3D11	BioLegend	Unconjugated^b^
HLA-F (KIR3DS1-Fc)	KIR3DS1	–	R & D Systems	Unconjugated^a^
HLA-E	NKG2A	3D12	BioLegend	Unconjugated^b^

^a^Chimeric protein binding was detected using a polyclonal anti-human IgG (Fcγ-specific)-PE conjugated secondary antibody (BioSciences)

^b^3D11 and 3D12 binding was detected using a polyclonal F(ab’)_2_ anti-mouse IgG-APC conjugated secondary antibody (eBioscience)

Results are presented as the MFI of cells stained with mAbs/Fc chimeric protein to each aNKR ligand versus FMO/isotype control/secondary antibody alone staining. The MFI of staining for individual ligands was reported as background subtracted FMO, isotype control or secondary antibody alone MFI. The control used to background subtract staining for each condition is indicated in Table 2.

**Table 2:** Mean fluorescence intensity (MFI) of activating NK receptor ligand staining on K562, .221 and CEM.NKr.CCR5 cells by specific antibodies and chimeric proteins

Ligand ID	Cell Stained											
	K562				.221				CEM.NKr.CCR5			
	MFI^a^	Range MFI	Ctl^b^	Background subtracted MFI	MFI	Range MFI	Ctl	Background subtracted MFI	MFI	Range MFI	Ctl	Background subtracted MFI^g^
Panel 1												
ULBP-1	895	852, 935	586^c^	309	389	382, 396	413^c^	− 24	276	271, 281	201	75^c^
ULBP-2/5/6	3779	3685, 4905	503^d^	3276	93.5	85, 123	79^d^	14.5	115	101, 127	24^d^	90
ULBP-3	1480	1219, 1895	749^d^	731	396	374, 437	379^d^	17	191	164, 223	159^d^	32
MIC-A	1065	923, 1192	357^d^	707	710	587, 1212	196^d^	514	170	164, 183	156^d^	16
MIC-B	786	784, 793	204^d^	514	667	468, 877	113^d^	554	107	94, 118	40^d^	67
Panel 2												
CD48	1044	788, 2347	169.2^d^	874.5	43,843	30,131, 54,430	299^d^	43,544	10,684	5081, 16,047	90^d^	10,594
CD80	8980.5	7293, 10,778	527^d^	8453	45,424	25,478, 65,004	526^d^	44,898	469	425, 807	449^d^	20
CD86	355	317, 367	264^d^	90	55,878	53,432, 56,184	22^d^	55,856	85	52, 112	67^d^	17
CD112	3211	2485, 3923	296^d^	2915	1.5	0.1, 12.1	1.6^d^	− 0.12	544	470, 675	270^d^	234
CD155	6187.5	5368, 6955	135^d^	6052	70.8	67, 73	51^d^	19.8	45	42, 54	28^d^	18
Panel 3												
ICAM-1	5087	4656, 11,162	497^d^	4590	16,875	11,852, 22,761	612^d^	16,875	203	185, 220	117^d^	86.5
ICAM-2	54,429	50,356, 57,335	227^d^	54,201	60,331	44,271, 78,093	379^d^	59,952	61,416	47,186, 87,979	103^d^	61,313
NKp30	1173	1152, 1217	157^e^	1016	182	165, 209	208^e^	− 25	160	116, 189	78^e^	82
NKp44	668	619, 836	157^e^	511	406	373, 469	208^e^	145	297	194, 350	78^e^	218
NPp46	302	165, 324	157^e^	145	299	197, 450	208^e^	91	268	209, 314	78^e^	190
HLA Panel												
HLA-F (3D11)	10,690	7197, 13,867	1252^c,e^	9438^f^	5032	4905, 6218	236^c,e^	4796^f^	1710	1608, 1881	189^e^	1521
HLA-F (KIR3DS1-Fc)	9630	6437, 12,587	416^c,e^	9219^f^	8093	5789, 9287	236^c,e^	7856^f^	2861	2578, 3600	187^e^	3380
HLA-E	280	208, 359	12^e^	267	171	99, 250	2.5^e^	169	477	435, 690	48^e^	428

^a^Median of 6 replicates, MFI – mean fluorescence intensity

^b^Ctl = Control staining

^c^Control staining where control was an isotype control

^d^Control staining where control was fluorescence minus one

^e^Control staining where control was a fluorochrome conjugated secondary antibody alone

^f^The isotype control was used for background correction

^g^Average of 6 replicates

### Statistical analysis

Statistical analysis was performed using GraphPad Prism version 6 (GraphPad Software, Inc., La Jolla, CA). Mann-Whitney tests were used to assess the significance of differences in the MFI of each aNKR ligand’s cell surface expression level compared to their respective control staining conditions. Kruskal-Wallis tests with Dunn’s post tests were used to determine the significance of differences in the background subtracted MFI generated by staining K562, .221 and CEM.NKr.CCR5 for each of the cell surface aNKR ligands tested. The distribution of the MFI of ligand expression is reported as median (range) of the replicate values. *P*-values less than 0.05 were considered significant.

## Results

### The MFI of aNKR ligand expression on K562, .221 and CEM.NKr.CCR5 cells

**Fig. 1:**
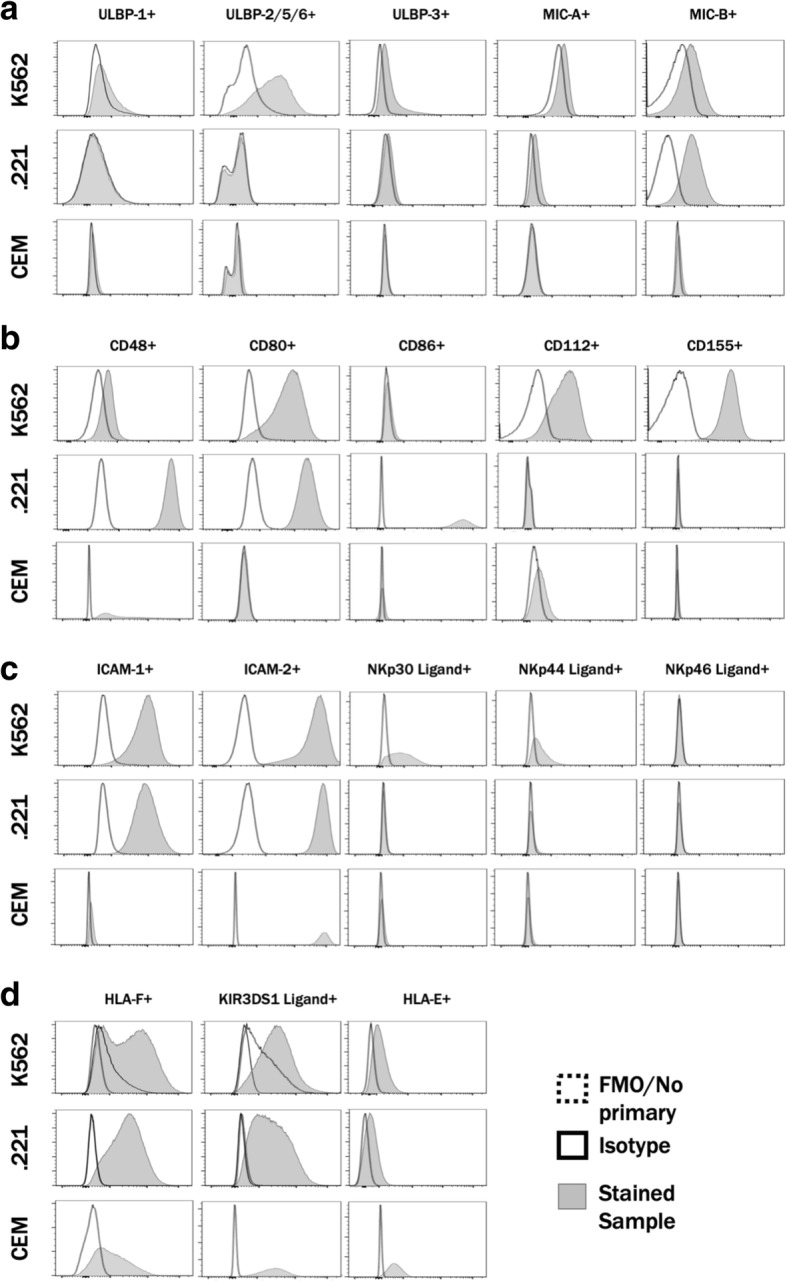
Staining K562, .221 and CEM.NKr.CCR5 cells with monoclonal antibodies (mAbs) and chimeric proteins specific for activating NK cell receptors (aNKR). Shown are examples of flow cytometry plots generated by binding fluorochrome conjugated mAbs, chimeric proteins or unconjugated mAbs with fluorochrome conjugated secondary antibodies specific for aNKRs to K562 (top rows in each panel), .221 (middle rows in each panel) and CEM.NKr.CCR5 (bottom rows in each panel) cells. Staining was done using Panel 1 (**a**) Panel 2 (**b**), Panel 3 (**c**) and Panel HLA (**d**) mAbs or chimeric protein reagents. Grey histograms represent staining with mAbs or chimeric proteins binding to aNKR. White histograms represent staining with fluorescence minus one, isotype control or secondary antibody alone controls. For information on which control was used for staining with each mAb or chimeric protein see Table 2

**Fig. 2:**
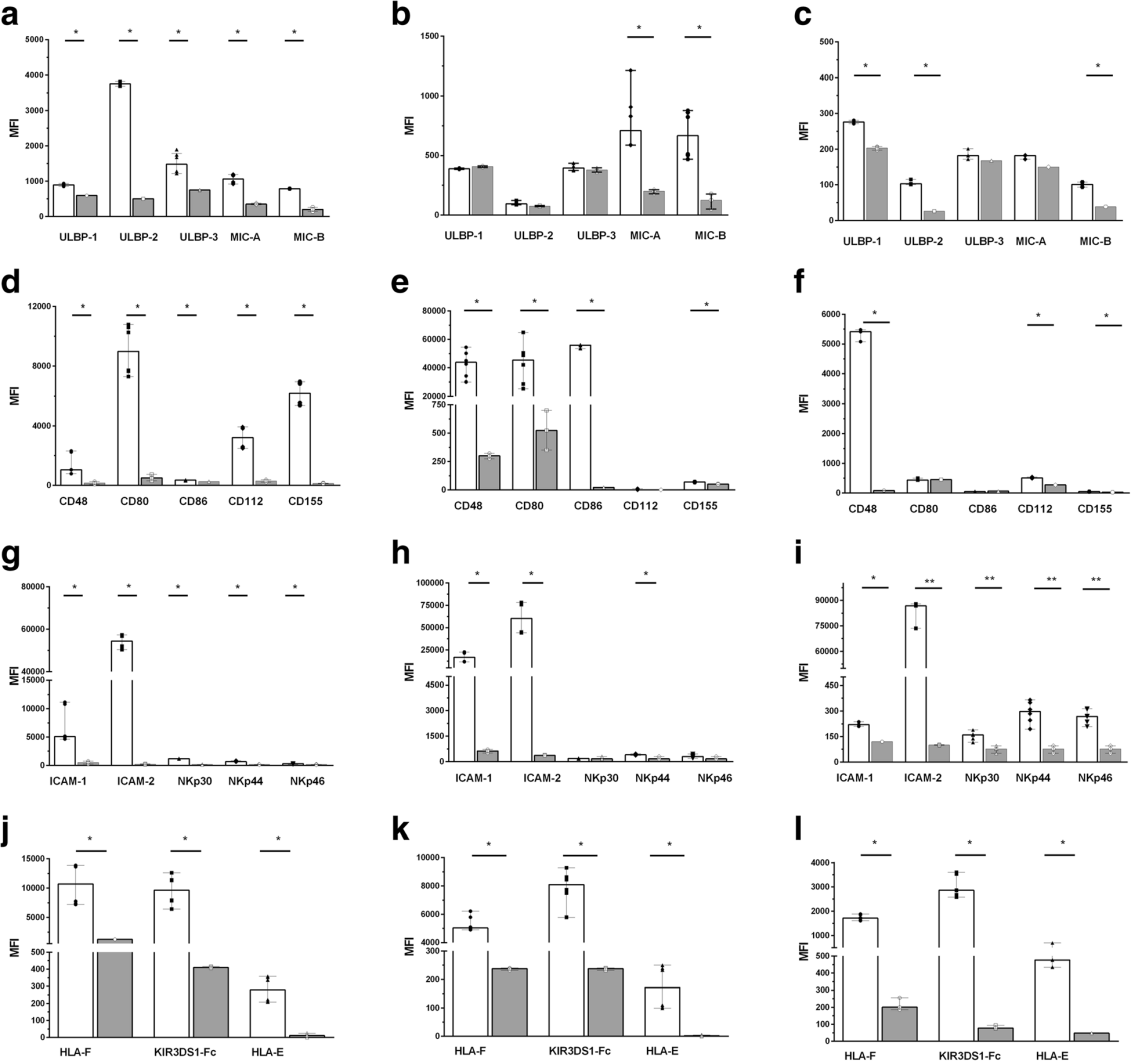
Mean fluorescence intensity (MFI) generated by staining K562, .221 and CEM.NKr.CCR5 cells with monoclonal antibodies (mAbs) and chimeric proteins to activating natural killer cell receptor (aNKR) versus their respective background controls. The y-axis shows the MFI of binding generated by using (**a**-**c**) Panel 1, (D-F) Panel 2, (**g**-**i**) Panel 3, and (**j**-**l**) Panel HLA anti-aNKR ligands reagents to stain to K562 (**a**, **d**, **g**, **j**), .221 (**b**, **e**, **h**, **k**) and CEM.NKr.CCR5 cells (**c**, **f**, **i**, **l**). White bars depict the MFI of staining with Panel A, B, C or HLA reagents while grey bars show the MFI of staining with an isotype control for ULBP-1 staining, fluorescence minus one controls for the other Panel A, Panel B and Panel 3 anti-ICAM-1 and anti-ICAM-2 antibodies and secondary antibody alone for all other aNKR ligand specific reagents. Bar height and error bars represent the median and range for the data set. Each data point represents one of three to six replicates. Significant differences are indicated by a line joining the observations being compared. (*) = *p*-values < 0.05 and (**) = *p*-values < 0.01

We hypothesized that differences in aNKR ligand levels expressed on K562, .221 and CEM.NKR.CCR5 cells could explain their differential abilities to stimulate NK cells. To address this, we determined the MFI of aNKR ligand expression by staining these cell lines with mAbs and chimeric proteins specific for these ligands and analyzing results by flow cytometry. The MFI of ligand staining was compared to that generated by the controls for each of the ligand specific reagents. Additional file 1: Figure S1 shows the gating strategy used to detect live singlet K562, .221 and CEM.NKr.CCR5 cells. Figure 1 shows examples of the histograms generated by staining K562, .221 and CEM.NKr.CCR5 cells with mAbs and chimeric proteins specific for aNKR ligands versus their FMO/isotype/secondary antibody controls. Table 2 and Fig. 2 show the median MFI obtained by staining these cell lines with the mAbs/chimeric proteins specific for aNKR ligands in Panels 1, 2, 3, and HLA versus their FMO/isotype/secondary antibody alone controls. Table 2 also shows the uncorrected, background corrected and control condition MFI staining levels of aNKR ligands on these three cell lines.

All the mAbs and chimeric proteins in the Panels 1, 2, 3 and HLA stained K562 at above background levels (Fig. 1a-d, top panels and Fig. 2a, d, g and j, *p* < 0.05 for all, Mann-Whitney tests). Many of these reagents also stained .221 at above background levels. Exceptions to this occurred for staining .221 cells with anti-ULBP-1, -ULBP-2/5/6, -ULPB-3 and -CD112 mAbs and with NKp30-Fc and NKp46-Fc chimeric proteins (Fig. 1a-d middle panels and Fig. 2b, e, h and k, *p* < 0.03 for all conditions that stained .221 cells at levels significantly above background, Mann-Whitney tests). The mAbs and chimeric proteins that stained CEM.NKr.CCR5 at levels above background were anti-ULBP-1, -ULBP-2, -MIC-B, -CD48, -CD112 and -CD155 mAbs and all the reagents in Panels 3 and HLA (Fig. 1a-d, lower panels and Fig. 2c, f, i and l, *p* < 0.05, Mann-Whitey tests).

### Comparison of the background corrected MFI of staining of aNKR ligand expression on K562, .221 and CEM.NKr.CCR5 cells

**Fig. 3:**
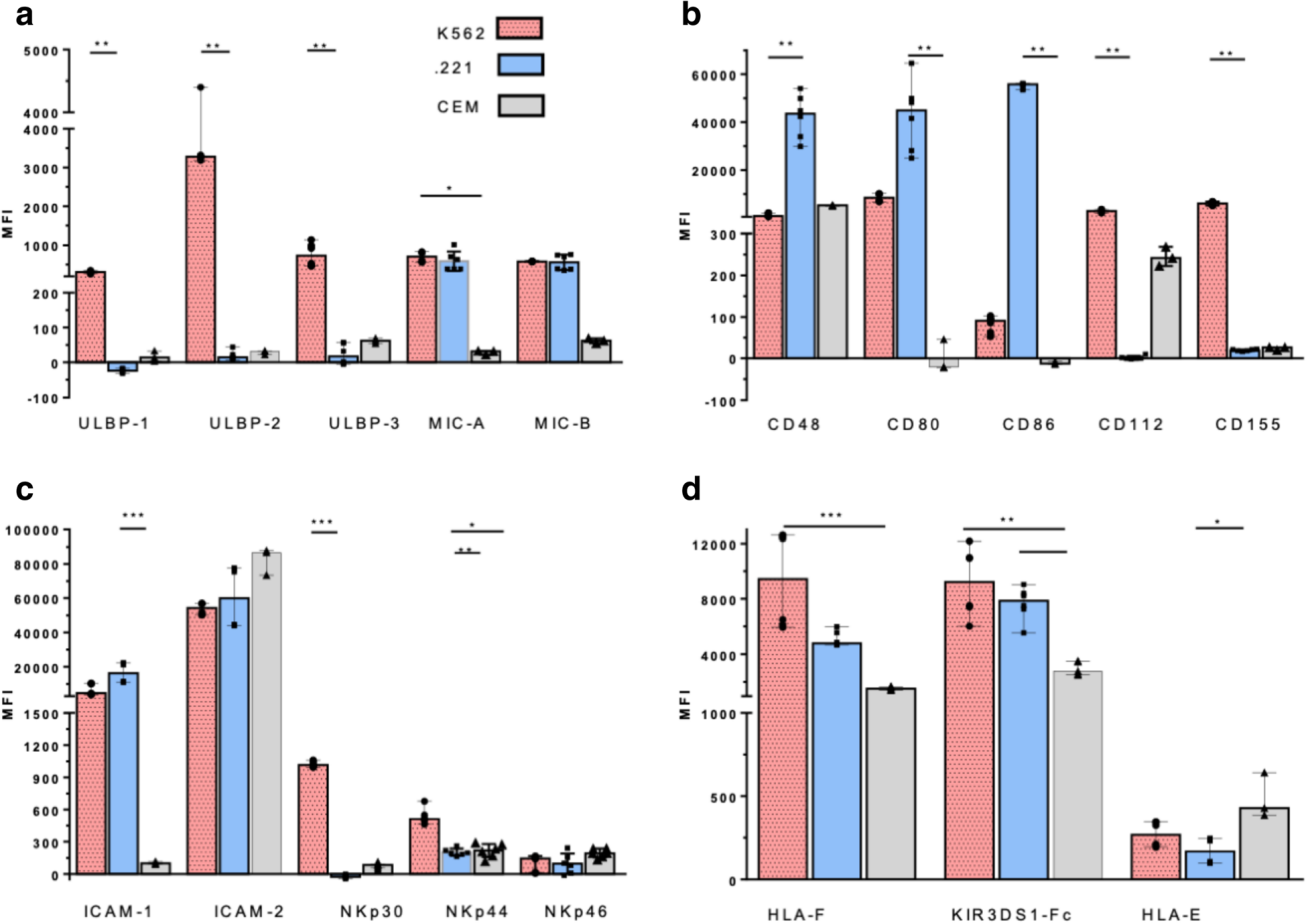
Comparison of the mean fluorescence intensity (MFI) generated by monoclonal antibodies (mAbs) and chimeric protein binding to activating natural killer cell receptor (aNKR) ligands on K562, .221 cells and CEM.NKr.CCR5 cells. The MFI of aNKR ligand expression on K562 .221 and CEM.NKr.CCR5 cells detected by (**a**) Panel 1, (**b**) Panel 2, (**c**) Panel 3, and (**d**) Panel HLA anti-aNKR ligand reagents is represented on the y-axis. Bar height and error bars represent the median and range for the data set. Each data point represents one of three to six replicates. Significant differences are indicated by a line joining the observations being compared. (*) = *p*-values < 0.05, (**) = *p*-values < 0.01 and (***) = *p*-values < 0.001

Figure 3 compares the background corrected MFI values generated by mAb/chimeric protein staining of K562, .221 and CEM.NKr.CCR5 cells. Most of the mAbs/chimeric proteins stained these three cell lines with MFIs that were significantly different from each other (*p* < 0.034 for all, Kruskal-Wallis tests). The only exceptions to this were for staining these three cell lines with anti-ICAM-2 and the NKp46-Fc chimeric protein.

Dunn’s post tests showed that K562 expressed ULBP-1, ULBP-2/5/6, ULBP-3 with higher background corrected MFIs than did .221 cells (*p* < 0.01 for all, Table 2 and Fig. 3a). The MFI of background corrected MIC-A and MIC-B cell surface staining was not significantly different on K562 and .221 cells, while K562 expressed the MIC-A ligand at a higher MFI than did CEM.NKr.CCR5 cells (*p* < 0.05, Table 2 and Fig. 3a). Staining with anti-CD48, -CD80 and -CD86 mAbs was highest on .221 cells and lowest on CEM.NKr.CCR5 cells. Observations that achieved statistical significance were lower expression of the CD48 ligand on K562 than .221 cells (*p* < 0.01, Table 2 and Fig. 3b). The CD112 and CD155 ligands were expressed at higher levels on K562 than .221 cells (*p* < 0.01 for both, Table 2 and Fig. 3b). The ICAM-1 ligand was expressed with a higher MFI on .221 than on CEM.NKr.CCR5 cells (*p* < 0.01) while the ICAM-2 ligand was expressed on all three cell lines at high levels that were not significantly different from each other (Table 2 and Fig. 3c). The ligands for NKp30 and NKp44 were expressed at a higher MFI on K562 than on .221 (*p* < 0.001 and *p* < 0.01, respectively) and the NKp44 ligand levels were higher on K562 than CEM.NKr.CCR5 cells (*p* < 0.05, Table 2 and Fig. 3c). KIR3DS1-Fc and mAb 3D11 bind HLA-F. HLA-F was present at a higher MFI on K562 than on CEM, NKr.CCR5 cells (*p* < 0.001, for both) and staining with KIR3DS1-Fc revealed a higher MFI of HLA-F expression on .221 than on CEM.NKr.CCR5 cells (*p* < 0.05). On the other hand, HLA-E was present at a higher corrected MFI on CEM.NKr.CCR5 cells than on .221 cells, while both K562 and .221 cells expressed HLA-E at background corrected MFIs that did not differ significantly from each other (Table 2 and Fig. 3d).

Together, these results show that K562 cells expressed higher levels of several of the ligands for the aNKRs NKG2D and NCRs than did .221 and CEM.NKr.CCR5 cells. K562 differed from .221 by having lower expression levels of the CD48 ligand. K562 also has lower levels of CD80 and CD86 ligand expression that did not achieve significance when Dunn’s post tests were applied. However, the CD80 and CD86 ligand expression levels were significantly higher on .221 cells than on either K652 or CEM.NKr.CCR5 cells when results for .221 and K562 were compared using Mann-Whitney tests (*p* = 0.002 and 0.005, respectively). The median MFI of background corrected aNKR ligand expression generated by staining CEM.NKr.CCR5 cells with the mAbs/chimeric proteins in panel 1, 2 and 3 was low for 4 of the ligands tested (MFI < 234). Only anti-CD48 and anti-ICAM-2, stained CEM.NKr.CCR5 cells with a high MFI that was readily distinguishable from background staining. This cell line also expressed higher levels of HLA-E than did K562 and .221 cells and expressed above background levels of HLA-F that were lower than those observed on K562 and .221 cells.

## Discussion

In this report we screened three cell lines that are frequently used to test NK cell functionality for expression of a panel of aNKR ligands. K562 and .221 cells are both HLA-null cell lines that should have a similar ability to abrogate NK cell inhibitory signals mediated by HLA binding to their iNKR. iNKR-HLA interactions determine NK cell education status and functional potential [60, 61]. Previous work showed that these HLA-null cell lines induced differential patterns of IFN-γ secretion, CCL4 secretion and CD107a expression by NK cells, suggesting that expression patterns for aNKR ligands may differ between these 2 cell lines and explain differential activation patterns. We found that K562 and .221 expression levels did not differ significantly for MIC-A, MIC-B, ICAM-2, HLA-E and NKp46 ligands. K562 expressed significantly higher levels of the NKG2D ligands ULBP-1, ULBP-2/5/6 and ULBP-3 and the ligands for NCRs NKp30 and NKp44. K562 differed from .221 by having significantly lower expression levels of CD48 and lower levels of CD80, and CD86 that did not achieve statistical significance using Dunn’s post tests. On the other hand, the NK resistant cells line CEM.NKr.CCR5, often used as a target cell in ADCC assays, expressed most of the aNKR ligands tested at low expression levels, with the exception of the ligands for CD48, ICAM-2, KIR3DS1 (HLA-F) and NKG2A/C (HLA-E).

Although prior studies have partially characterized the expression profiles of ligands to aNKRs on K562 and .221 cells, to our knowledge this has not been done for the CEM.NKr.CCR5 cell line that differs from its parent CEM cell line by being resistant to direct NK cell cytolysis [25, 62]. In this study, we included a larger and more comprehensive panel of mAbs/chimeric proteins detecting aNKR ligands than have previously been investigated.

The receptors implicated in triggering NK cell mediated cytolysis include NKG2D and the NCRs [62–65]. The ligands for NKG2D include ULBP-1, ULBP-2/5/6, ULBP-3, MIC-A and MIC-B [65]. NKG2D is a C type lectin receptor that associates with signaling molecules such as DAP10/KAP10 to initiate the cascade of events leading to cytolysis [17, 66–68]. The NCRs include NKp30, NKp44 and NKp46, among others [65]. They associate with different tyrosine kinase activating motifs bearing signal transducing polypeptides to mediate activating signals [31, 32]. The characterization of the ligands for these NCRs is incomplete, which is why chimeric proteins based on these receptors are used to probe for the presence of their ligands on target cells. Blocking the interaction of NCRs and NKG2D with these ligands also reduces cytolysis mediated by NK cells, highlighting the role of NKG2D engagement in NK cell killing [62].

In our previous studies comparing the functional profile induced in NK cells by K562 and .221 cell stimulation, we found that while both HLA null cell lines were able to induce CD107a expression, the functional NK subsets that included CD107a expression contributed to a higher proportion of the total NK cell response when stimulated by K562 than by .221 cells [16]. The higher expression level of NKG2D and NCR ligands on K562 than on .221 cells reported here and the lower levels of expression of other ligands for aNKR such as CD48, CD80 and CD86 would be consistent with an aNKR ligand profile that favors NK cell stimulation towards CD107a expression, which is a marker for degranulation, a step in the pathway toward cytolysis. NKG2D signaling has been shown to play a crucial role in NK cell cytotoxic granule polarization, degranulation, and cytotoxicity [69]. The ability of K562 to induce signaling through this pathway may explain why this cell line preferentially stimulates NK cell degranulation, rather than cytokine or chemokine secretion [16]. Unfortunately, no antibody currently exists that can discriminate between ULBP-2, − 5, and − 6 and we are incapable of determining which combinations of these ligands are expressed on K562. However, it is possible that K562 cells express ligands for all three of these receptors, in addition to ligands for ULBP-1 and -3. The consequence of this may be induction of more potent signaling through NKG2D of K652 than .221 cells, which only express MIC-A and MIC-B with a modest MFI. Moreover, expression of NKp30 on NK cells has been correlated with both perforin expression and degranulation. Engagement of this aNKR by K562 may also contribute to their induction of degranulation [70]. CD112 and CD155 bind to DNAM-1 [18, 28, 29]. Thus, K562 has a ligand profile that does not only stimulate NK cells through NKG2D, NKp30 and NKp44 but also through DNAM-1.

Our finding that .221 cells express the ULBP-1, ULBP-2/5/6 and ULBP-3 ligands for NKG2D at a low MFI that is not much above background levels is in line with a report by Pende et al. [62]. Our findings differ for MIC-A, which we found was expressed over background by .221 cells while Pende et al. found it not to be expressed over background. These discrepant results may be due to differences in the reagents used to detect the NKp30 ligand or to other technical issues relating to staining and analysis methods. These findings are consistent with previous reports that ULBP ligands are preferentially expressed on K562 and with the observation that K562 cells express the tumor ligand B7-H6, which is a ligand for NKp30 [21, 62].

The higher expression levels of CD48, CD80, CD86 and ICAM-1 on .221 than K562 cells may explain why .221 cells stimulated a higher frequency of functional NK cells, particularly those secreting IFN-γ and CCL4 than did K652 [16]. When K562 expressed a higher MFI of aNKR ligands than .221 cells, as was in the case of CD112, CD155 and HLA-F, the differences were more modest than were the between-cell differences in CD48, CD80, CD86 and ICAM-1 expression on .221 versus K562 cells. The elevated expression level of these ligands on .221 cells may provide these cells with a more potent activating signal. Engagement of the aNKR 2B4 by its ligand CD48 on .221 has been reported to induce low levels of IFN-γ production and concurrent signaling through 2B4; signaling through other aNKRs can drive high levels of cytokine and chemokine production [9, 20]. Although, the exact NKRs that recognize the co-stimulatory molecules CD80 and CD86 have not been identified, expression of these ligands on target cells can trigger NK cell-mediated cytolysis [50]. Together, the ligands that are expressed on .221 are capable of engaging receptors that are important for cytokine and chemokine production, which may explain why .221 cell stimulate larger numbers of IFN-γ and CCL4 secreting NK cells than do K562 cells [16].

Our findings confirm that HLA-E, the ligand to the aNKRs NKG2E and NKG2C and the iNKR, NKG2A, on NK cells, is expressed by K562 and .221 cells with a low MFI that was nevertheless above background staining levels [71]. On the other hand, CEM.NKr.CCR5 cells express cell surface MHC-I antigens and higher HLA-E levels than do K562 and .221. Stable cell-surface expression of HLA-E requires binding to one of a set of nonamer peptides derived from the leader sequences of MHC-Ia molecules or HLA-G, which are absent on the HLA-null .221 cell surface [72, 73]. It is unlikely that HLA-F expression by K562 and .221 cell lines contributes to HLA-E expression as the signal sequence of HLA-F does not have a nonamer peptide able to bind HLA-E [55]. However, the HLA-E peptidome was recently shown to be less restricted than previously thought. HLA-E can also bind to an array of self-peptides in the absence of HLA class I signal peptides, permitting its stable expression and induction of NK cell cytotoxicity [74, 75]. In addition, HLA-E is also capable of presenting EBV-derived peptides, such as BZLF1, which would be expected to be present in the EBV transformed .221 cell line [76, 77]. HLA-E was recently shown to present a cytomegalovirus derived signal peptide important in driving the expansion of adaptive-like NKG2C+ NK cells [78]. HLA-E/BZLF1 complexes are poorly recognized by NK cells and it is likely that HLA-E molecules presenting non-canonical self, make greater contributions to .221-induced NK cell activation [76].

The non-classical MHC-I antigen, HLA-F, is a KIR3DS1 ligand, which is expressed by K562, .221 and CEM.NKr.CCR5 cells [56–58]. HLA-F has also been reported to interact with KIR3DL2 and KIR2DS4 [79]. Although the interaction of HLA-F with KIR3DL2 was confirmed by others, its interaction with KIR2DS4, which is structurally related to KIR3DL2 due to a gene conversion event has not been confirmed [56, 80]. HLA-F is also expressed on HIV infected cells [56]. Other investigators did not observe HLA-F on K562 [56, 57]. Here, we used both the mAb 3D11 and KIR3DS1-Fc to stain K562 cells for cell surface HLA-F. Both reagents generated concordant results for the presence of HLA-F on this cell line.

*KIR3DS1* homozygotes were more frequent in a population of HIV exposed seronegative than in HIV susceptible individuals and *KIR3DS1* homozygotes remained uninfected for longer time intervals despite HIV exposure than those with other *KIR3DL1/S1* genotypes, suggesting that KIR3DS1 HLA-F interactions may provide protection from HIV infection [81, 82]. The global distribution of KIR3DS1 varies from one population to another [83, 84]. For example, it is rare in sub-Saharan African populations [83]. It is interesting to speculate on whether HLA-F/KIR3DS1 or /KIR3DL2 or possibly /KIR2DS4 combinations can influence HIV control mediated by NK cells and whether this could account for between-individual or -population differences in HIV susceptibility or the rate of HIV disease progression.

For the purpose of this study, the ligands analyzed were included on the basis of their ability to stimulate NK cell responses through the engagement of aNKRs. However, it is important to consider that several of these ligands are capable of engaging both aNKRs and iNKRs. CD112 and CD155, which signal through the activating DNAM-1, can also bind to the iNKR, T cell immunoreceptor with immunoglobulin and ITIM motifs (TIGIT) [85, 86]. While both DNAM-1 and TIGIT are widely expressed on NK cells, the affinity of CD155 for TIGIT is greater than for DNAM-1 and TIGIT expression can reduce DNAM-1/CD155 interactions in a dose-dependent manner [87–89]. TIGIT has also been shown to compete with DNAM-1 for the binding of CD112. Furthermore, when transfected into the NK cell line YTS, TIGIT greatly limits NK-mediated cytotoxicity by disrupting cytotoxic granule polarization [89, 90]. Considering this, it is possible that CD112, which is exclusively expressed on K562, and CD155 which is expressed at higher levels on K562 than .221 cells contributes more to NK cell inhibition than activation and may be an additional reason why K562 activates a smaller fraction of NK cells, compared to .221 [16]. Another aNKR ligand, HLA-E, similarly contributes to both NK cell activation and inhibition. HLA-E binds to the CD94/NKG2 family of NK cell receptors, which includes the activating NKG2E and -C and the inhibitory NKG2A and -B NKRs [53, 54]. Interactions between NKG2A, which is expressed on the majority of NK cells, and HLA-E have been shown to predominate over interactions with NKG2C and surface expression of HLA-E is sufficient to rescue those cells from lysis by NKG2A^+^ NK cells [53, 54, 91]. Despite this, work assessing NK cell stimulation by autologous HIV-infected CD4 T cells, which express HLA-E, found that expression of NKG2A on NK cells was associated with improved activation [92]. It is plausible that, while interactions between HLA-E on .221 and NKG2A on NK cells can tune down NK cell activation, signaling through the other aNKR engaged by .221 ligands can compensate for this inhibitory input.

CEM.NKr.CCR5 cells expressed few aNKR ligands. Ligands for CD48 and ICAM-2 were present on this cell line as well as low levels of ligands for NCRs. The interaction of ICAM-2 with its ligand may contribute to the formation of NK cell CEM.NKr.CCR5 conjugates [65]. Less is known regarding the consequences of CD48 ligand expression. It is interesting to speculate that the presence of few other cell surface ligands for aNKR contributes to the resistance of this cell line to direct NK cell cytolysis in the absence of an antibody bridging target and effector cells [25]. Pende et al., tested the CEM.NKr.CCR5 parental cell line, CEM, for cell surface expression of ULBP-1. ULBP-2, ULBP-3 and MIC-A and found that CEM cells expressed ULBP-2 and ULBP-3 [62]. The absence of these aNKR on CEM.NKr.CCR5 is suggestive that the process of selecting for CEM.NKr resistance to direct NK cell cytolysis led to loss of several ligands for aNKR [25].

## Conclusion

The two HLA-null cell lines K562, .221 and the ADCC target cell CEM.NKr.CCR5 differed in their expression of ligands to aNKR. The data presented here provide a systematic assessment the stimulatory potential of three cell types commonly used to study NK cell activation. The different aNKR ligand expression profiles of K562, .221 and CEM.NKr.CCR5 are associated with the induction of qualitative differences in the NK cell responses to these cell lines. CEM.NKr.CCR5 cells, that are resistant to direct NK cell killing express few ligands for aNKR. This work provides a basis for examining the specific contribution of each ligand-aNKR pair to different stages of NK cell activation.

## Additional file

Additional file 1: **Figure S1.** Gating strategy. Gating strategy identifying live, singlet, (A) K562 and (B) .221 and (C) CEM.NKr.CCR5 cells. Forward and side scatter plots were used to gate on cells as indicated by the outlined area of each left-hand plot. From these cells, singlets were gated on as shown in the outlined area of the middle panels. From the singlet population live cells were gated on as indicated by the outlined area in the right-hand plots. (TIF 411 kb)

## Abbreviations

221: 721.221
ADCC: Antibody dependent cellular cytotoxicity
aNKR: Activating natural killer cell receptors
CD: Cluster of differentiation
DNAM-1: Leukocyte adhesion molecule DNAX accessory molecule-1
FBS: Fetal bovine serum
HLA: Human leukocyte antigen
ICAM: Intercellular adhesion molecule
IFN-γ: Interferon-γ
ILC: Innate lymphoid cell
iNKR: Inhibitory natural killer cell receptor
IU: International unit
KIR: Killer immunoglobulin-like receptors
LFA-1: Lymphocyte function-associated antigen 1
mAb: Monoclonal antibody
MFI: Mean fluorescence intensity
MHC-I: Major histocompatibility complex class I
MIC: Major histocompatibility complex class I related chain
NCR: Natural cytotoxicity receptors
NK: Natural killer
NTB-A: Natural killer-T-B cell antigen
RT: Room temperature
TIGIT: T cell immunoreceptor with immunoglobulin and ITIM motifs
ULBP: UL-16 binding protein
