# Supplementary figures and images for: Expression of ligands for activating natural killer cell receptors on cell lines commonly used to assess natural killer cell function
Source: BMC Immunol. 2019 Jan 29;20:8. doi: 10.1186/s12865-018-0272-x (PMC6352444; doi:10.1186/s12865-018-0272-x)

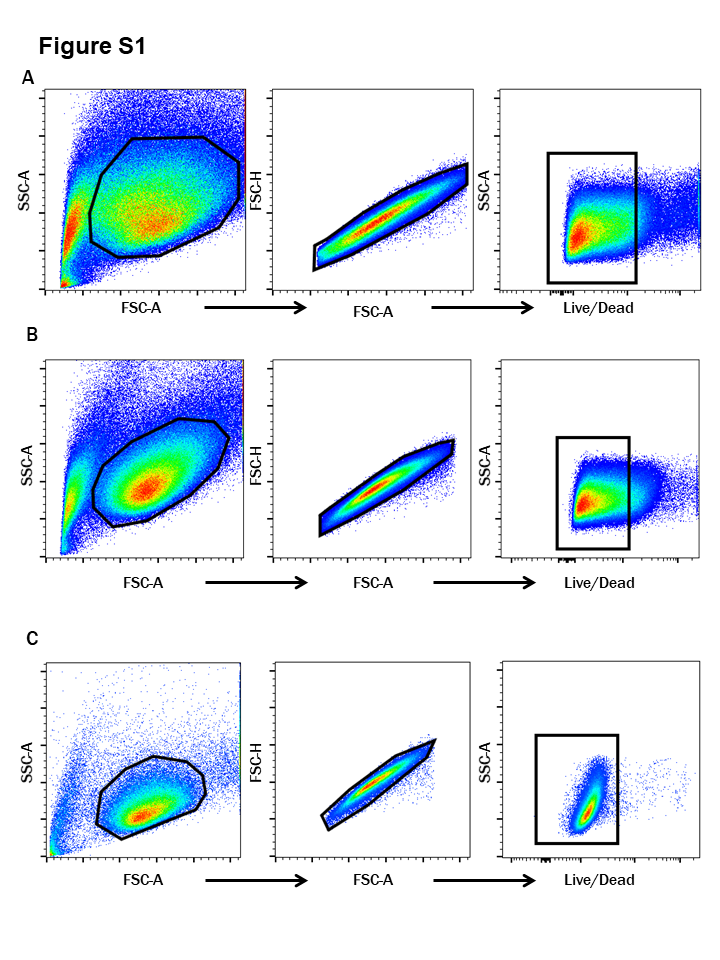

Supplement: Supplementary file 1 — Figure S1. Gating strategy. Gating strategy identifying live, singlet, (A) K562 and (B) .221 and (C) CEM.NKr.CCR5 cells. Forward and side scatter plots were used to gate on cells as indicated by the outlined area of each left-hand plot. From these cells, singlets were gated on as shown in the outlined area of the middle panels. From the singlet population live cells were gated on as indicated by the outlined area in the right-hand plots. (TIF 411 kb) [file 12865_2018_272_MOESM1_ESM.tif]
